# Supplementary material for: Is vaginal delivery of a fetus in breech presentation at an extremely preterm gestational age associated with an increased risk of neonatal death? A comparative study
Source: PLoS One. 2021 Oct 20;16(10):e0258303. doi: 10.1371/journal.pone.0258303 (PMC8528279; doi:10.1371/journal.pone.0258303)
Supplement: Attachment — Submitted filename: Responses to the reviewers.docx [file pone.0258303.s001.docx]

PONE-D-20-34255
Is vaginal delivery of a fetus in breech presentation at an extremely preterm gestational age associated with an increased risk of neonatal death? A comparative study

| Reviewer #1: | Our responses |
| --- | --- |
| How should preterm breech fetus be planned to deliver ? This is an intersting topic. Given that it is not a frequent situation, all experiences are welcome to counsel women.  In fact, this mansucript is incomplete and flawed by many methodologic bias that need to be adressed before publication. Moreover, the text is not well structured because there are an amount of data and the results should be separated.  Therefore, the manuscript IS VERY SPRAWLING (breech versus cephalic, factors associated with mortality in vaginal delivery, explanation of risk of entrapment ....) Results of this manuscript could be interesting if the authors performed several changes so that readers could undertsand and compared the results to their own data.  See below the main comments | See comments below |

| The aim of the study is to compare Planned vaginal delivery and planned caesarean delivery for preterm breech delivery. Therefore, the aim IS NOT to study if preterm breech delivery has an increased risk of perinatal death.  One may suppose that breech fetus are at an increased risk of perinatality. The increased risk observed in these fetuses coule be linked to antenatal or underlying disorders that may be associated with the breech presentation and so not solely due to the mode of delivery. In that way, it is intersting to compare cephalic versus breech presentation. However it is not your topic.  When you councel a women, you are not going to councel her by saying : as compared to cephalic presention, breech presentation is at increased risk, so let's perform a CS. You are going to explain , by comparing the MODE of delivery for preterm breech delivery, the safer is ... or is not..  Moreover, by excluding congenital malformation, birthweight < 500g, medical termination of pregnancy and fetal detah, if the ratio cephalic/breech is not equally distributed as compared to the selected population, selection bias is highly probable. As I could read : 69,1% of preterm cephalic had CS versus 31,25 % of preterm breech . This important difference can not be a random ratio. It reflects selection bias that may favor cephalic presentation  Therefore, in my opinion group 2 is not necessary. Only data on the overall neonatal mortality rate of preterm cephalic fetus is necessary to give to have an overview of the practice in your center | We agree with reviewer # 1 and thank him for his comments. As group 2 was not really necessary, we deleted all the data concerning it, which simplified our manuscript, indeed a little sprawling. . |
| --- | --- |
| 2. Period study : authors should explained why they ended the inclusion to the years 2015 | The data available to us for this study did not allow us to go beyond 2015. |
| 3. Main problem of external validity that need to be adressed - the group 1 :CS rate during labor 3,5% This is a french study. I could compare this results with 2 recent french studies : Kayem et al (26-30 WP) (20%) and lorthe (24-34 WP) et al (18,4%)  How can the authors explain this important difference ? External validity is questionable  ? | The main difference with our study is the gestational age, which is earlier in our study. Before 28 weeks, the prevalence of uterine infection is high and labor is rapid. In addition, the risks and difficulties of cesarean section probably make obstetricians more reluctant to do it . |
| 4. As written in the text, the authors argued for a high experience in breech delivery. Therefore in the method, authors should give the selection criteria for a trial of labor | Our study was carried out in a university maternity which performs around 5,000 deliveries per year. The selection criteria for a trial of labor has been the subject of a protocol and regular publications. Adjaoud S, J Gynecol Obstet Biol Reprod 2017 ; 46 :445-8. |
| 5. Period study. Between 1997-2015, several pediatrics improvments have been performed . Authors performed a chi-square test by comparing neonatal mortality betwwen 1997-2005 and 2005-2015. I am not convinced by this method that is very biased and unprecised.Therefore to be sure that there is no time varying effect, authors should at least give :  - A run line series plot for the 2 groups - And if you can Check for trends and then you can perform a correlation test (not useful for some statisticien), or computing the variance of the 2 groups if they are independent (not reasonable assumption, because it is the same hospital ?) or Vector Auto-regression model if the 2 groupes are dependant | We agree and segmented the study period into successive 5-year periods, then performed a trend test that was not significant. We have thus noted in the text:  The risk of death in the delivery room for breech PVDs remained stable around 12 % over our 19-year study period : 18, 11, 20 and 10 % during 5 years consecutive periods (chi2 for trend, p = 0.62). |
| 6. The authors found an increased risk of neonatal morbidity in planned vaginal delivery. What we want to know is : why ? Is it solely the mode of delivery or the obstetrical underliyng condition or selection bias ?  Missing important data : reason for CS DELIVERY ? Reason for preterm delivery ? According to recent french data , (delorm et al. 2016), cause of preterm delivery could be a prognotic factors (such as low birthweight...) . As I can read in the table 1, in the group 3 (PCD), 32,5% had spontaneous preterm delivery. What was the reason to perform CS ? Therfore the main problem is the allocation to PVD or PCD ? This is not well described in the manuscript Due to its retrospective nature the authors should be very precise to describe the reason for the mode of delivery | We are fully aware that the two groups are not comparable and we told it in the “results” and “discussion” sections: the “PVD” group exclusively includes spontaneous births (100.0%) while the PCD group includes only 32.5% spontaneous deliveries. Limiting the comparison to spontaneous deliveries would have raised an insufficient statistical power. Obviously, the fetuses of the PCD group - born in a situation of induced prematurity - had a higher gestational age by a few days, but they were more fragile, due to more frequent IUGR and far lower birth weight  Adjusting the results for gestational age and birth weight seemed to us to be the best solution to take these differences into account. In these conditions, the adjusted OR reflects for us the risk specific to the mode of delivery  Finally, the non significant excess risk associated with vaginal delivery (ORa 2.6 [0.8 - 9.3]) seems to remain interesting due to :  - Our results go in the same direction that many other studies of the medical literature that we mentioned in the manuscript  - Our study deals with is a rare but worrying situation for obstetricians  - Situation in which many countries have not issued recommendations despite the gradual accumulation of results in favor of a cesarean section  About « the reason why" the PVD would be deleterious,  our study provides original and often poorly reported data on what happens before leaving delivery room, with head entrapments that concern 9 of the 14 deaths and cord prolapse 3/14, risks known to be associated with breech presentation in vaginal delivery.  To answer your questions and improve our discussion, we have added the following sentence  “In these vaginal births with death in delivery room, head entrapments concern 9/14 and cord prolapse 3/14: that is, 12 out of 14 were associated with risks known to be related to breech presentation in vaginal delivery. Thus, mortality in the delivery room appears to be associated mainly with an excess number of complications that are strongly related to breech presentations (21). Although these risks have been mentioned by others (14,16,17), our study is original in highlighting the cause of this excess mortality in the delivery room. |
| 7- As written in the results, group 1 and 3 are very different. Therefore, all these confounding variables need to be addressed in a regression analysis | We specified that the method of calculating the adjusted OR was logistic regression in the Material and Method section  “We used logistic regression to take into account prespecified factors usually associated with perinatal mortality: gestational age, birthweight, fetal gender” |
| 8- Discrepencies in the data : Latter deliveries but lower Birthweight in PCD ... This may reflect either selection bias or codding bias or different policies for planned dleivery that need to be explained. | We addressed this point in the answer to question 6 |
| 9- Duration of labor : less than 60min in 50%. Authors should explained how they defined the beginning of the labor. | The beginning of the labor was arbitrarily defined by the arrival in the delivery room. We precised it in the M&M section  Duration of labor (from arrival in delivery room to delivery) |
| 10- missing data, that need to be adressed in group 1 and 3 : type of breech (franck, non franck, footling), clinical chorioamnionitis, use of tocolysis, the use of magnesium sulphate, economic status, admission after labor onset (in utero transfer), sex ratio, use of antibiotics, detailed of breech vaginal delivery management | We added supplementary data that reviewer asked for, if available :   - Maternal caracteristics related to economic status : BMI, (smokers was already related) - Sex ratio - Analgesia/anesthesia during labor   Clinical choriamnionitis was not available (histologic choriamnionitis was already mentioned). About Magnesium sulfate, we started tu use it in 2015, so no fetus was concerned by this prophylactic treatment. |
| 11- Regression analysis should be detailed. In the table 2 (with some form typo), I could read adjusted OR. However I could not see either in the method nor in the result section the explanation of this adjustment. Factors of death have been studied only in the PVD group? Usually to perform adjused analysis, you should select factor according : *confounding factors *no colliders * avoiding unnecessary covariate * avoiding multicolinearity  And then you run a regression |  |
| 12. Table 3-4 : This table adresses the factors associated with mortality in PVD. This is very intersting and could be a rational for another manuscript.  In conclusion, due to the amount of data, I suggested to the authors :  - To study mode of delivery on breech fetus : PVD vs PCD. To perform a classical analysis of the causal framework between mode of delivery and mortality  - Or to to study only group 1 and to perform an analysis (by regression, or a detailed descriptive analysis) to understand the neonatal mortality after PVD | We agree with this proposition. So we have therefore limited our analysis to the comparison of the PVD and PCD breech groups. |
|  |  |
| Reviewer #2: |  |
| Pierre and colleagues compare neonatal outcomes along three groups of singleton preterm deliveries: planned breech vaginal delivery, planned cephalic vaginal delivery, planned breech cesarean delivery.  The authors did a nice job in the discussion explaining how their research adds to the literature (consideration of spontaneous vs induced preterm delivery; distinction between death in delivery room vs NICU). The introduction would be strengthened by similarly setting up a distinction between what is known from prior literature and what this study adds. | In the introduction, we added that the causes of the possible excess of neonatal death in case of trial of labor are unclear :  “Most authors report that a trial of vaginal delivery for fetuses in breech presentation before 28 weeks is accompanied by an increase in neonatal mortality (7,10–15), although a few have not reported it in extremely preterm infants (16,17). The cause of this possible excess of neonatal mortality is mostly not specified. Few countries recommend a routine cesarean delivery in this situation (18); others make no recommendations at all about mode of delivery (19,20).”  At the end of the introduction, we also specified:  (…). In order to better understand the possible excess mortality in case of trial of labor, we wanted to differentiate the mortality occurring in the delivery room from that occurring in the neonatal intensive care unit. |
| My biggest concern is the difference in findings between the bivariate analysis and adjusted analysis, how these findings are explained, and the conclusions drawn from them. The bivariate analysis demonstrates that neonatal mortality in the breech PVD group was twice that of the cephalic PVD group (OR = 2.5) and almost triple that of the breech PCD group (OR = 2.9). However, as reported, there are significant differences in gestational age, birthweight, and rates of induction between the breech vaginal and breech c-section groups. After adjusting for GA and birthweight, there is no longer a significant difference in morbidity between the breech vaginal and breech c-section groups. Gestational age is the known key factor in outcomes for premature deliveries, so this is not unexpected. | We slightly changed our discussion to highlight that, even if not significant, the results of our study are interesting.  - They go in the same direction - and in the same proportions - as most of the other studies carried out so far,  - They specify that by studying mortality more precisely, this excess is due to mortality in the delivery room, mainly by head entrapment (which had not been specified so clearly so far). |
| In the abstract, the highlighted results should be the adjusted ORs. The conclusions drawn should be that differences in neonatal mortality between breech vaginal and breech c-section groups were no longer significant after controlling for gestational age and birthweight (although differences between cephalic PVD and breech PVD groups persist). | We finaly reported the adjusted OR in the abstract, stating that it was not significant. This does not change - for us - the interest of the abstract, which specifies that the increased mortality is only observed in the delivery room.  “Although not significant after adjustment, neonatal mortality in the breech PVD group was more than twice that of the breech PCD group (19.5 vs 7.5%, *P*=0.026, ORa=2.6, 95% CI 0.8-9.3, NNT=8). This higher neonatal mortality in the breech PVD group was exclusively associated with a significative higher risk of death in the delivery room 12.4 vs 0.0% *P*=0.001, OR not calculable, NNT=8.” |
| It is also unclear if the adjusted OR are adjusted for variables other than GA and birthweight? This should be described clearly. Other clinically relevant variables like induction, parity, receipt of steroids, presence of chorio, should be evaluated in bivariate analysis and considered for inclusion in the adjusted logistic regression. | We agree with this comment. In Material and Methods section, we told more precisely how was our adjustment was undergone  “We used logistic regression to take into account prespecified factors usually associated with perinatal mortality: gestational age, birthweight, fetal gender”  It seemed unreasonable to adjust for many more factors with the size of our series (113 vs 80). |
| The discussion should comment on why the authors think the GA is higher in the PCD groups but the birthweight is lower. | We mentioned this in the discussion section  First, the groups compared retrospectively are far from identical in terms of gestational age and birth weight (aged a few days extra, but very often severely stunted in the PCD group), and the adjustment may not take into account all the differences between the groups. This is common to all retrospective studies of this type (14, 15, 19). |
| Regarding decisions to deliver vaginally vs by c-section, it would be helpful to explain in the methods how PVD vs PCD is determined from record review. Was there any hospital protocol to make this decision, or did the attending obstetrician use their personal clinical judgement? A context about the number of different providers making these decisions would also be helpful. Since this is an observational retrospective study, this study is limited by non-measured factors (potential confounds) that made healthcare providers decide to deliver some of these breech fetuses vaginally and others by c-section. This should be added to the discussion. | In our center and so far, there is a protocol for vaginal delivery in breech presentation that has been published (it provides for the performance of maternal pelvimetry around 37 weeks) (ref 1 and 2). Before 37 weeks and up to now, trial of labor for breech fetuses is considered possible, including at the youngest gestational ages. In the PCD group, the decision of cesarean section before labor is taken on the existence of a contraindication to the vaginal route such as the existence of a multi-scarred uterus, FHR anomalies before labor, or a maternal refusal of a trial of labor. In this group, the existence of FHR anomalies during labor led, however, to perform 4 cesarean sections during labor (3.5%)   1. 1. Adjaoud S, J Gynecol Obstet Biol Reprod 2017; 46: 445-8. 2. Michel S et al. Evaluation of a decision protocol for type of delivery of infants in breech presentation at term. Eur J Obstet Gynecol Reprod Biol. 2011;158(2):194-8 |
| The term “global” neonatal mortality is confusing. I recommend changing it to “total” and defining it clearly as death during delivery + death in NICU.  Was NICU followup stopped at 28 days (definition of neonatal mortality) or continued though discharge? | We agree and named the global mortality "total mortality" with changes in the whole manuscript.  The principal endpoint was total neonatal defined as i) death during delivery plus ii) death in the neonatal intensive care unit (NICU). ~~We distinguished the deaths in the delivery room from those in the (NICU)~~.  Regarding the mortality after transfer to NICU (n = 8/22 (PVD) and n = 6/6 PCD), it occurred before 28 days in all cases of the PVD group (8/8) and in 4 of the 6 deaths of the PCD group. However, it seemed fairer to us to speak of intra-hospital mortality without taking into account this 28-day delay after birth, especially for such premature and immature children. |
| Table 3: What is “Gestational age 25 weeks” compared to? Is this 25/0-25/6 compared to >26 weeks? The title is confusing. | We agree. In the last row of this table 3, we have replaced  ~~«  Gestationnal age > 26 »~~ by « Gestational age 25 weeks (vs 26 or 27) |
| Table 4: The title is confusing. Categories with <5 items should be compared using Fisher’s Exact, not Chi Squared. In “25 SA”, “26 SA”, etc, what does “SA” mean? | We agree. We corrected and used Fisher's exact test comparing two groups when necessary. One of the differences was no longer significant (the decrease in the risk of death with gestational age in the event of retention on the last head, p = 0.20) and we have specified this in the Results section. For us, this does not alter the demonstration made by our paper. |
|  |  |
|  |  |

Reviewer #2: 
