# Supplementary material for: Is vaginal delivery of a fetus in breech presentation at an extremely preterm gestational age associated with an increased risk of neonatal death? A comparative study
Source: PLoS One. 2021 Oct 20;16(10):e0258303. doi: 10.1371/journal.pone.0258303 (PMC8528279; doi:10.1371/journal.pone.0258303)
Supplement: Attachment — Submitted filename: Responses to reviewers.docx [file pone.0258303.s002.docx]

Review Comments to the Author

| Reviewer #1: Some comments. Thanks you for giving me to review the revised manuscript. I am convinced that the manuscript could deserve publication. However to improve the clarity, the understanding and in fact external validity some comments need to be addressed. Some data needs to be published especially the excess of neonatal death due to head entrapment for example. In my opinion the authors should just simplified some analysis, the table 2 and the process of selection of women. |  |
| --- | --- |
| 1. As the primary outcomes is the combined neonatal death in delivery room and in NICU, there is a little discrepencies between the sentences in the introduction and in the material and method  In order to better understand the possible excess mortality in case of trial of labor, we wanted to differentiate the mortality occurring in the delivery room from that occurring in the neonatal intensive care unit. The principal endpoint was total neonatal defined as i) death during delivery plus ii) death in the neonatal intensive care unit (NICU). | We took account of this suggestion in the Introduction section and replaced  ~~we wanted to differentiate the mortality occurring in the delivery room from that occurring in the neonatal intensive care unit.~~  By : total neonatal mortality was separated as i) death during delivery plus ii) death in the neonatal intensive care unit (NICU). |
| I read the manuscript for a second time and I try to be a "new reader". I may not understand fully the objectives of the authors.  Firstly , The authors want to provide data to counsel women when : (i) preterm delivery is occuring (ii) fetus is in breech. Therefore, the group of planned CS should not be a mixture of induce/spontaneous labor and real planned CS. Women with induction of labor should be in the planned vaginal birth In fact, women with spontaneous preterm labor in breech or with premature preterm rupture of membrane or with medically indication birth should be offered 2 options: PVD or PCD = PVD (spontaneous or induction) vs PCD So the authors should clearly explained the 26 women with spontaneous labor in the PCD group. I may imagine that it is women with spontaneous preterm labor and the obstetrician may have chosen to perform CS. However, should these women include in PCD group? May be yes in latent phase of labor, no in active phase of labor Secondly the difficulty is that the group of PVD might be a group with worse prognosis. Therefore, the reader need to understand why the obstetrician chose PVD or PCD | We fully understand this remark and our two populations are not comparable, as we have clearly expressed in the Results and Discussion sections. It would have been preferable to compare the effects of the route of delivery only in fetuses having spontaneous labor, that is to say in 113 fetuses (PVD) vs 26 fetuses (PCD), but the numbers in the PCD group would have been very weak. This group is made up of 26 fetuses out of 139 presenting breech before 28 WA (18.7%) for which the obstetrician chose to perform a cesarean section at the very beginning of labor for a reason that is not always known.  We brought up this point in the discussion:  « Furthermore, the reason why 26 of the 139 fetuses (18.7%) whose labor was spontaneous had a PCD and not a PVD in our study was not always known. Our results, however, are consistent with other studies “ |
| 3. Reason for neonatal morbidity should be given as no difference was observed between APGAR, chorioamniotis, IVH, hemorrhage, leukomalacia ?? What is the effect of antenatal corticosteroids? | We agree with this remark. It is unlikely that corticosteroid therapy would be responsible for the excess mortality in the delivery room, since 90.3% of newborns in the PVD group had received corticosteroid therapy (vs 96.3%, p = 0.11 ). On the other hand, it is likely that the number of Apgar score <5 and the number of pH <7 were minimized due to missing values in the PVD group. |
| 4. Statistical analysis: I am not convinced by the adjusted OR because some factors might be effect modifier One simple possibility to understand the factors associated with neonatal mortality is to perform stratified analysis and mantel haenzel test to distinguish confounding factors and effect modifier: especially for gestational age, use of antenatal corticosteroids, gender, birthweight (500 – 600 – 700 – 800 or < 3°percentile vs no), by providing crude and adjusted M-H OR. In fact multiple logistic regression is somewhat not useful with small sample size and collinearity (birthweight and gestational age ++). | We understood your comments and performed a Mantel Haenzel test for each factor that could have acted as « effect modifier » rather than « confounding factor » None of the tests showed a significant interaction (no significant variation in the effect depending on whether the factor was present or not) Increased mortality related to PVD (vs PCD) Ajusted on \| Adjusted OR MH \| Interaction test (p); Gender \| 2.9 [1.1 – 7.7] \| 0.58; Antenata corticosteroid therapy \| 2.7 [1.0 – 7.1] \| 0.71; Gestationnel age > 25 wks \| 2.2 [0.8 – 5.9] \| 0.31; Birth weight < 700 g \| 7.1 [1.9 - 25] \| 0.62; Birth weight < 900 g \| 3.7 \|1.3 – 10] \| Not realisable |
| 5. Can the authors explained the increased death in delivery room with cord prolapse ? Cord prolapse during expulsive stage or during active phase labor. As only 3.4% of women in PCD had CS , can the authors explain the management according to cord prolapse? | Cord prolapse occured in 4 fetuses of our series of 113 fetuses with PVD (3.5%), which is consistent with the excess cord prolapse observed in breech presentation. For the three cases where the death occurred, the duration of labor was too short to perform a cesarean section (5, 10 and 20 minutes) |
| 6. The significance of this excess of mortality disappeared after adjusting for gestational age, birthweight and gender at delivery This sentence should be deleted as statistical significance is trivial when analyzing neonatal death. In 2016, the American Statistical Association released a statement in The American Statistician warning against the misuse of statistical significance and P values Not all values inside are equally compatible with the data, given the assumptions. The point estimate is the most compatible, and values near it are more compatible than those near the limits | We deleted the sentence "The significance of this excess of mortality disappeared after adjusting for gestational age, birthweight and gender at delivery" as well as two parts of sentences that referred to this adjustment. |
| In your manuscript, whatever the OR, there is a clear association between mode of delivery and neonatal death. The only question is why ? In fact the authors should clearly explained the management during labor, and the other comment previously written (reason for PCD, effect modifier or confounding, discrepancies between no diiference for pH and other morbidity while neonatal death in delivery room only occurred after PVD) | We answered this question in the discussion part :  “This shows that the increased neonatal mortality for breech PVD infants we observed was associated exclusively with the excess of mortality in the delivery room. In these vaginal births with death in delivery room, head entrapments concern 9/14 and cord prolapse 3/14: that is, 12 out of 14 were associated with risks known to be related to breech presentation in vaginal delivery. Thus, mortality in the delivery room appears to be associated mainly with an excess number of complications that are strongly related to breech presentations (21).”  This difference in mortality rate is not accompanied by a difference in neonatal morbidity, which indicates that once the risk of death have been passed in the delivery room, the neonatal prognosis is identical depending on the mode of delivery. |
| 7. Our study was carried out in a university maternity which performs around 5,000 deliveries per year. The selection criteria for a trial of labor has been the subject of a protocol and regular publications. Adjaoud S, J Gynecol Obstet Biol Reprod 2017 ; 46 :445-8. I disagree with the authors. In this article, the protocol is for term breech trial and not for preterm delivery….. I cited : « Women were included if they had given birth during the study period to a singleton child at term, in either breech or vertex presentation. Multiple pregnancies, medically indicated terminations of pregnancy, and in utero deaths were excluded from the study » | We agree. We wanted to indicate that the monitoring of labor and delivery and the eligibility for trial of labor are the subject of validated written protocols in cases of breech at term, but being evaluated in situations of prematurity, as in this work |
| 8; Table 2 : typo 10 and 12% instead of 0,10 et 0,12. I am not convinced with the p value for mean pH Item asphyxia should be given i.e : ph < 7 or base excess or lactate | We added a line in Table 2 about those three items gathered together. No significant difference appeared (11.5 % in PVD group vs 10.0% in PCD group) |
| Reviewer #2: Overall, the authors did a very nice job with revisions and the paper is stronger and more statistically accurate. |  |
| In the abstract, the conclusion states that “For deliveries between 25+0 and 27+6 weeks' gestation, vaginal delivery in breech presentation is associated with a significantly higher risk of death in the delivery room.” Again, I would be careful with using this language of “significance” when differences in mortality were significant in the adjusted analysis. | We agree and deleted “significantly” in the abstract |
| I understand the rationale of not adjusting for many variables given the small sample size, but I do think that parity (history of vaginal delivery) is a clinically very important variable when it comes to head entrapment risk and successful vaginal delivery. If you choose not to adjust for parity, I recommend specifically mentioning that as a limitation in the discussion. | We understand this remark. We explored the role of parity in Table 3. In PVD, no link between nulliparity and death could be found our study. |
| In the third paragraph of the results, I recommend specifying that the initial results you describe are “bivariate analysis” or “unadjusted analysis.” | We agree and wrote “unadjusted analysis showed that” in this paragraph. |
| Adding a column to table 2 with adjusted OR (with a footnote explaining what is was adjusted for) may help readers better understand the difference in OR/significance for mortality in bivariate analysis vs adjusted analysis | We agree and added a specific column to Table 2 |
